# The potential impact fraction of population weight reduction scenarios on non-communicable diseases in Belgium: application of the g-computation approach

**DOI:** 10.1186/s12874-024-02212-7

**Published:** 2024-04-14

**Authors:** Ingrid Pelgrims, Brecht Devleesschauwer, Stefanie Vandevijvere, Eva M. De Clercq, Johan Van der Heyden, Stijn Vansteelandt

**Affiliations:** 1 Department of Chemical and Physical Health Risks, Sciensano, Rue Juliette Wytsman 14, 1050 Brussels, Belgium; 2 Department of Applied Mathematics, Computer Science and Statistics, Ghent University, Krijgslaan 281, S9, BE-9000 Ghent, Belgium; 3 Department of Epidemiology and Public Health, Sciensano, Rue Juliette Wytsman 14, 1050 Brussels, Belgium; 4 Department of Translational Physiology, Infectiology and Public Health, Ghent University, Salisburylaan 133, Hoogbouw, B-9820 Merelbeke, Belgium

**Keywords:** Non-communicable diseases, Overweight, g-computation, Potential impact fractions, Health policy, Health impact assessment

## Abstract

**Background:**

Overweight is a major risk factor for non-communicable diseases (NCDs) in Europe, affecting almost 60% of all adults. Tackling obesity is therefore a key long-term health challenge and is vital to reduce premature mortality from NCDs. Methodological challenges remain however, to provide actionable evidence on the potential health benefits of population weight reduction interventions. This study aims to use a g-computation approach to assess the impact of hypothetical weight reduction scenarios on NCDs in Belgium in a multi-exposure context.

**Methods:**

Belgian health interview survey data (2008/2013/2018, *n* = 27 536) were linked to environmental data at the residential address. A g-computation approach was used to evaluate the potential impact fraction (PIF) of population weight reduction scenarios on four NCDs: diabetes, hypertension, cardiovascular disease (CVD), and musculoskeletal (MSK) disease. Four scenarios were considered: 1) a distribution shift where, for each individual with overweight, a counterfactual weight was drawn from the distribution of individuals with a “normal” BMI 2) a one-unit reduction of the BMI of individuals with overweight, 3) a modification of the BMI of individuals with overweight based on a weight loss of 10%, 4) a reduction of the waist circumference (WC) to half of the height among all people with a WC:height ratio greater than 0.5. Regression models were adjusted for socio-demographic, lifestyle, and environmental factors.

**Results:**

The first scenario resulted in preventing a proportion of cases ranging from 32.3% for diabetes to 6% for MSK diseases. The second scenario prevented a proportion of cases ranging from 4.5% for diabetes to 0.8% for MSK diseases. The third scenario prevented a proportion of cases, ranging from 13.6% for diabetes to 2.4% for MSK diseases and the fourth scenario prevented a proportion of cases ranging from 36.4% for diabetes to 7.1% for MSK diseases.

**Conclusion:**

Implementing weight reduction scenarios among individuals with excess weight could lead to a substantial and statistically significant decrease in the prevalence of diabetes, hypertension, cardiovascular disease (CVD), and musculoskeletal (MSK) diseases in Belgium. The g-computation approach to assess PIF of interventions represents a straightforward approach for drawing causal inferences from observational data while providing useful information for policy makers.

**Supplementary Information:**

The online version contains supplementary material available at 10.1186/s12874-024-02212-7.

## Background

By affecting almost 60% of adults and nearly one in three children in the European Region, excess body weight is the fourth most common risk factor for NCDs, after high blood pressure, dietary risks, and tobacco use [1]. In Belgium, as in many high-income countries, average body mass index (BMI) has continuously increased over the past decades among both children and adults [2]. According to the most recent Belgian Health Interview Survey (BHIS) conducted in 2018, 48% of the adult population suffered from overweight (BMI > 25) and 14% from obesity (BMI > 30), compared to respectively 41% and 11% in 1997 [3]. Tackling obesity is therefore one of the greatest long-term health challenges in Belgium, such as in other countries, and is vital to successfully achieve the Sustainable Development Goals with regards to the reduction of premature mortality from non-communicable diseases [4].

Assessing the contribution of excess weight status as risk factor for NCDs and evaluating the potential health impact of policies for the prevention of overweight presents certain challenges and methodological issues, especially when using observational and cross-sectional data. To capture the association between a risk factor exposure and a health outcome, typical approaches in epidemiological studies use linear or logistic regression models, which estimate the differences between outcomes associated with a change in the risk factor exposure. This approach, relying on stratum-specific estimates, is however limited because it is not informative on how the burden of disease might change by modifying the risk factor exposure in the population. Furthermore, in the case of logistic regression models, interpretation of the obtained odds ratio is subtle because of non-collapsibility: it tends to move further away from 1 when adjusting for more and more variables, even in the absence of confounding [5]. The population attributable fraction (PAF), a concept introduced by Levin [6], is a measure widely used by epidemiologists to estimate the proportion of a disease attributable to a risk factor in a given population [7]. The PAF is typically calculated using the relative risk and the prevalence of the risk factor in the population and is often interpreted as the proportion of cases in the population avoidable if a particular risk factor was eliminated. The PAF based on Levin’s formula [6] was originally unadjusted for co-existing (risk) factors but methods such as adjusted and average attributable fraction (AAF) or attribution methods have been developed since to account for multi-causal situations (i.e. when a given disease is caused by more than one causal mechanism) [8–11]. However, for the PAF/AAF to have a valid causal interpretation, strong assumptions are required. This is because the excess cases seen in people with overweight need not all be “attributable” to overweight: they may not all be overweight-induced but rather the effect of other risk factors prevalent in those people [12, 13]. Unfortunately, those assumptions are often disregarded or misreported in articles [12]. In addition, the PAF assumes that there is an optimal intervention which completely eradicates the risk factor in the population which is often unrealistic because a part of the population will often continue to be exposed to the risk factor, even with the most effective intervention. The potential impact fraction (PIF), also called the generalized impact fraction, is another measure that allows to estimate the fractional reduction of cases that would occur from changing the current level of exposure in the population to some modified level [14]. The PAF and the PIF, both affected by the strength of the association between the disease and the risk factor as well as the prevalence of the risk factor, estimate the disease risk in the population in case of “complete withdrawal” and “partial reduction” of the exposure [15, 16]. The application of the traditional PAF or PIF for policymaking in this context is strongly limited by the rigors of complete elimination of the risk factor as well as the disadvantages of traditional methods based on standard regression models [7, 17].

To overcome those limitations, the use of causal inference methods has been suggested by several authors [18–23]. In particular, the g–computation approach (a model-based direct standardization) has the advantage that it can handle continuous risk factors and predict the causal impact of public health interventions on the population burden of disease, using cross-sectional data [18, 24, 25]. Unlike traditional regression models, the method allows the estimation of population parameters, where the population average causal effect is estimated as the difference in the health outcome that would have been observed in the population if there had been a specific intervention as opposed to no intervention (everything else remained equal). Those population intervention parameters allow determining which hypothetical intervention may have the greatest impact on the disease. The method requires to clearly specify the causal effect of interest and to explain all assumptions needed to identify this effect from the available data. This can be achieved using a directed acyclic graph (DAG) which is a graphical representation used to illustrate the hypothesized causal structure of the processes under study [23, 26]. Compared to standard analytic techniques, the method also enables modelling the impact of dynamic interventions, where different subjects can receive different levels of the exposure under study [27]. Although causal inference methods, and in particular the g-computation approach, have already been well described in the literature as a useful tool for assessing intervention effects and producing policy-relevant findings [28–30], their application in public health remains however limited [31]. In particular, g-computation has not yet been extensively used for studying the health impact of excess weight [32–34].

Other common methodological issues in observational studies aiming to evaluate the potential health impact of exposure-reducing interventions are related to the validity of self-reported data. Although a large body of literature already exists on methods to obtain more accurate surveillance data by correcting for measurement error related to self-reported data in health interview surveys, few epidemiologic studies use them in practice [35, 36]. The measurement biases are however not without consequence because when exposures are not valid, the PIF estimates may be severely biased.

This study aims to use a g-computation approach to quantify the effects of different population-based weight reduction interventions on important NCDs in Belgium in a multi-exposure context (taking into account lifestyle, metabolic, and environmental exposures). The research relies on cross-sectional data from the Belgian Health Interview Survey and Health Examination Surveys, addressing measurement bias due to self-reported health and anthropometric data through a random-forest multiple imputation method [37]. Additionally, this paper aims to provide a didactic application of the g-computation approach to assess PIF from cross-sectional data.

## Methods

### Study area, study population and data

The study area is the entire Belgian territory with a population of 11.6 million inhabitants in 2023. The study sample consists of 27 536 participants of different waves of the Belgian Health Interview Survey (BHIS 2008, 2013, and 2018) all aged 18 years and above. Additionally, it includes a subset of 1,184 participants who also took part in the Belgian Health Examination Survey in 2018 (BELHES 2018). The information from BELHES 2018 was primarily used to address measurement errors in self-reported health and anthropometric data.

The BHIS is a national cross-sectional population survey carried out every five years by Sciensano, the Belgian institute for health, in partnership with Statbel, the Belgian statistical office [38]. Data are collected through a stratified multistage, clustered sampling design and weighting procedures are applied to obtain results which are as representative as possible of the Belgian population [39]. In the BELHES, objective health information was collected among a random subsample of the BHIS participants. The BELHES included a short additional questionnaire, a physical examination, and the collection and analysis of blood- and urine samples. Details on the data collection are available in the BELHES publication [40].

Based on the geographical coordinates of the residential address of participants and using Geographical Information Systems (GIS), the dataset was further enriched with objective measures of the residential environment related to long-term exposure air pollution (Black carbon), green space (vegetation coverage in a 1 km buffer), and noise from road traffic (Lden, day–evening–night noise level).

### Abdominal obesity and non-communicable disease indicators

BMI and waist circumference were used as continuous variables, the latter to assess abdominal obesity. Four NCDs were considered: diabetes (type 1 & 2), hypertension, cardiovascular disease (CVD), and musculoskeletal (MSK) disease. The variables used to construct these indicators are displayed in Table 1.

**Table 1:** Construction of abdominal obesity and non-communicable disease indicators

Indicator	Variable description	
BMI	The measured imputed variable (using information from the BELHES and a randomforest multiple imputation method) was used instead of the self-reported variable	Based on the measured height (cm) and weight (kg)
Waist circumference		Based on the measured waist circumference (cm)
Diabetes		Based on fasted blood sugar (≥ 126 mg/dl), HbA1C (≥ 6.5%) or use of diabetes medication
Hypertension		Systolic blood pressure ≥ 140 mmHg or diastolic blood pressure > 90 mmHg or medication use for hypertension
Cardiovascular disease	Self-reported variable: «Suffered from the indicated disease in the past 12 months” for at least one of the following variables	-Stroke (cerebral haemorrhage, cerebral thrombosis) -Myocardial infarction -Angina pectoris -Other serious heart disease -Peripheral vascular disease
Musculoskeletal disease		-Low back pain -Neck pain -Osteoarthritis -Rheumatoid arthritis

### Socio-demographic and lifestyle indicators

The following variables were used to describe each participant’s socio-demographic status: *age (years)*, *sex (male vs female)*, *household composition (single, one parent with child(ren), couple without child(ren), couple with child(ren), other or unknown)*, *highest educational level in the household (No diploma/primary school, low secondary, high secondary, higher)*, *reported household income (quintiles), birth country (Belgian, Non Belgian EU, non-Belgian non EU)*, and *civil status (single, married, widow, divorced).* To describe the participant’s lifestyle, we used the variables:, *smoking status* (daily smoker, occasional smoker, former smoker or never smoked)*, indoor smoking (yes vs no), alcohol consumption,* and *level of physical activity (≥ *4 h sport or intensive training per week, < 4 h sport or light activities per week or sedentary behavior)*.* This last variable is based on the WHO indicator describing leisure time activity in the last 12 months [41], where sedentary behavior is defined as the complete absence of physical leisure activities. To assess alcohol consumption, we transformed the ordinal variable representing the average number of alcoholic beverages per week into a numeric variable. The numeric values assigned were as follows: 1 = Abstainers and occasional drinkers, 2 = 1 to 7 glasses, 3 = 8 to 14 glasses, 4 = 15 to 21 glasses, 5 = 22 + glasses. One glass stands for a “standard unit” which varies according to the type of alcohol (for example, 0,33 l beer, 0,125 l wine, 4 cl spirits, etc.).The reported household income, defined by the quintile distribution was also converted into a numeric variable. The binary variable *Indoor smoking* describes household where at least one person smokes inside the dwelling on most days.

### Environmental indicators

The selection of environmental factors in our study, including air pollution, green spaces, and noise, was guided by their well-established associations with NCDs. They represent a good proxy of the individual exposure since they were derived from the geographical coordinates of the survey participants' residential addresses.

Air pollution was assessed through the annual average of exposure to black carbon (BC). BC represents one of the most health-relevant components of particulate matter (PM) and is a valuable indicator to assess the health effects of air quality dominated by primary combustion particles [42]. BC exposure was obtained as a continuous grid through the Belgian Interregional Environment Agency (IRCEL – CELINE) which supervises the national monitoring system assessing air pollutant concentrations through a dense network of stations, and estimates local exposure through interpolation, taking into account land cover data in combination with a dispersion model [43, 44]. BHIS data of 2008, 2013, and 2018 were respectively linked to BC exposure data of 2010, 2013, and 2018. Exposure to green spaces was assessed based on CORINE Land Cover (CLC) data [45]. The vegetation coverage was obtained at the neighborhood level in a 1 km buffer around the respondent’s dwelling. This 1 km buffer of vegetation coverage is justified by the need to capture the immediate neighborhood environment that individuals are likely to interact with regularly and aligns with common practices in environmental epidemiology, where this scale is frequently used to assess the impact of neighborhood characteristics on health. Lifestyle factors, including physical activity and stress reduction, are influenced by the accessibility of these spaces in one's daily life. BHIS data of 2008, 2013, and 2018 were respectively linked to green space data of 2006, 2012, and 2018. Noise pollution, approached through the road traffic noise (Lden, day–evening–night noise level), was obtained from published noise maps, as required by the European Noise Directive (2002/49/EC) [46–48]. Noise data are created at the regional level and downloaded from the regional portals for environmental data [49–51]. BHIS data of 2008, 2013, and 2018 were respectively linked to noise data of 2016. Noise from the road traffic, is recognized as a significant environmental stressor associated with various health issues, including cardio-vascular diseases and a lower quality of life. The Lden metric provides a comprehensive measure of overall noise exposure and the 55 dB used cut-off aligns with the recommended WHO threshold, acknowledging the detrimental health impact above this threshold.

### Statistical analyses

All variables were described with their 95% confidence interval and the missing data pattern was displayed for the merged BHIS/BELHES dataset (additional file 1, 2, 3, 4, 5).

### Database compiling

In a first step, the measurement error related to self-reported height, weight, diabetes, and hypertension in the BHIS database was corrected based on the objective information included in the BELHES and using a random forest multiple imputation method. A MICE algorithm [52] was used to multiply impute the missing values of the merged dataset. The imputation model included all the variables of the dataset, including variables used in the weighting procedure associated with the survey sample design (province, number of persons by household, age, and sex). All missing values of the covariates included in the imputation models were imputed in the same process. Details on the application of this correction method in the BHIS is found in a previous publication [37]. The number of iterations of the random-forest multiple was set to 500 and the defined number of trees was set to 100. The convergence of the algorithm was monitored by plotting the mean and standard deviation of the synthetic values against the iteration number for the imputed BHIS data (Additional file 2). The number of imputations was limited to 10, which was found satisfactory: using infinitely many imputations instead of 10 was estimated to reduce the variance of the estimators by at most 1%. 

### Population impact fractions

In a second step, a g-computation approach was used in each of the 10 completed datasets to assess the PIF of four weight reduction scenarios:

1. a distribution shift where, for each person with overweight, a counterfactual weight was randomly drawn from the distribution of persons with a “normal” BMI (> = 18.5 and < 25)
2. a one-unit reduction of the BMI of people with overweight
3. a modification of the BMI of people with overweight based on a weight loss of 10%.
4. a reduction of the waist circumference (WC; cm) to half of the height (cm), among all people with a WC:height ratio greater than 0.5 [53].

The selection of these four scenarios aims to provide a comprehensive exploration of potential BMI reduction strategies and was guided by a combination of practical relevance and existing literature supporting their potential impact on health outcomes.

The impact of the first three scenarios on each NCD was evaluated for two target populations: people with overweight (BMI > 25) and people with obesity (BMI > 30). The fourth scenario was applied to the specified population.

The mean reduction in BMI was calculated for the first three scenarios, and the mean reduction in waist circumference was calculated for the fourth scenario. This was determined by subtracting the counterfactual BMI (or WC) under the intervention from the actual BMI (or WC) of each individual and then averaging these differences.

**Fig. 1:**
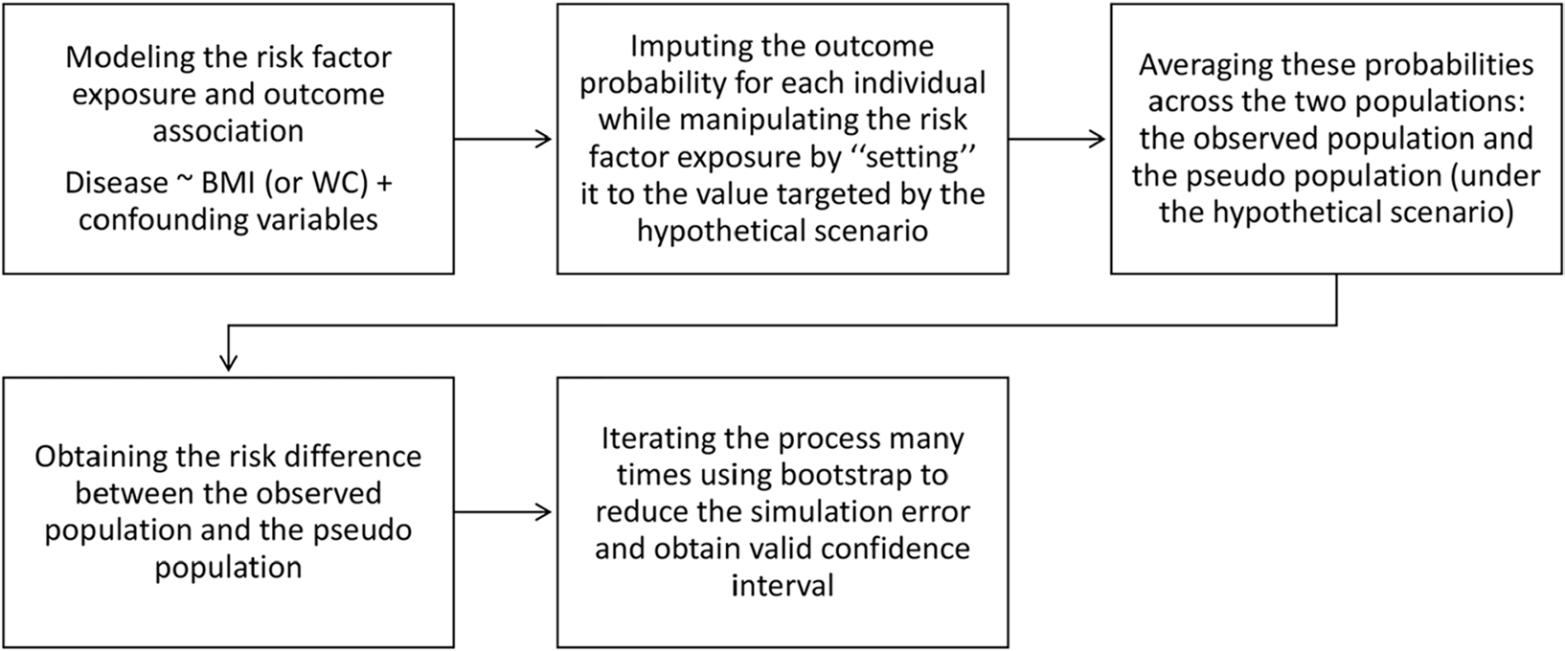
Steps of the g-computation approach

**Fig. 2:**
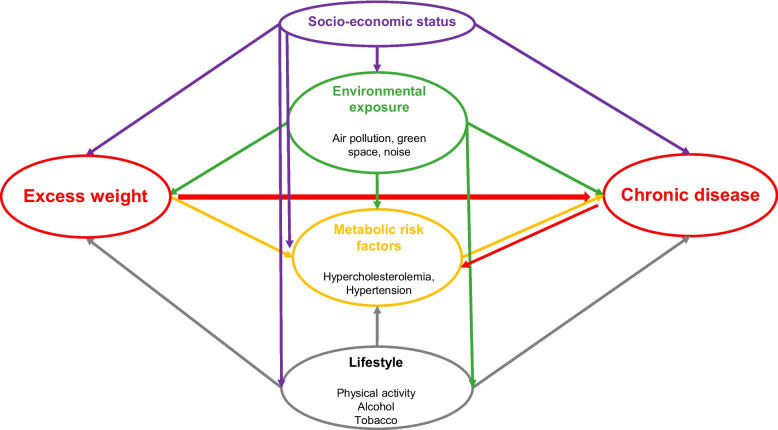
Directed acyclic graph of the causal association between excess weight and each of the four non-communicable diseases (diabetes, hypertension, cardiovascular disease, and musculoskeletal disease)

In each of the ten imputed datasets, standard errors of the PIF were obtained using 1000 nonparametric bootstrap samples. The imputation steps of the g-computation approach [28] are described in Fig. 1:

The association between excess weight and each NCD was modelled based on the “backdoor criterion” which is specific to the causal inference theory [54]. The DAG displayed in Fig. 2 illustrates the postulated causal structure of the association between excess weight and NCDs. Confounding factors such as socio-economic, environmental, and lifestyle factors influence this association. Excess weight affects NCDs through metabolic risk factors. Models were not adjusted for the metabolic risk factors (hypercholesterolemia and hypertension) since they were colliders or lying on the causal pathway between excess weight and the disease. Two logistic regression models were performed for the four NCDs considered. The first model included BMI and the second model included WC to assess the excess weight status. Models were adjusted for socio-economic, lifestyle, environmental factors, region, and year. Interactions were tested between BMI (and WC) and each of the covariates. The performance of the models was assessed by randomly splitting each of the ten imputed dataset into a training dataset (70%) and a test dataset (30%) and by evaluating the Area under the curve (AUC). The ten obtained AUC values were then averaged. In order to account for the potential indirect effect of BMI on chronic diseases through physical activity, sensitivity analyses were performed by fitting models without adjustment for physical activity.

The PIF of each scenario was calculated in each of the ten imputed dataset and results of the multiple analysis were pooled using the standard Rubin rules [55]. Standard errors of the prevalence estimates were obtained as the square root of the total variance (taking into account the within and between imputation variance and a correction factor for using 10 imputations). PIF were reported as percentage indicating the proportion of disease cases that would be avoided under the hypothetical weight reduction scenarios. The degree to which all the underlying assumptions required to draw a causal inference [56] (temporal ordering, exchangeability, no-interference, experimental treatment assignment, consistency, no model misspecification, no measurement error) is addressed in the Discussion section.

Statistical analyses were performed taking into account the survey sample design. The multistage sampling method was accommodated by incorporating weights, calculated to reflect the likelihood of being selected in the sample, based on the geographical stratification, the selection of clusters within each stratum, the choice of households within each cluster, and the selection of individuals within each household.

All analyses were fit and evaluated using the statistical software R, version 4.2.1 (R Development Core Team, 2006) and the “mice” package [57]. The R code used for the implementation of the G-computation to assess the PIF (for the diabetes example) is available in Additional file 6.

## Results

### Data description

**Fig. 3:**
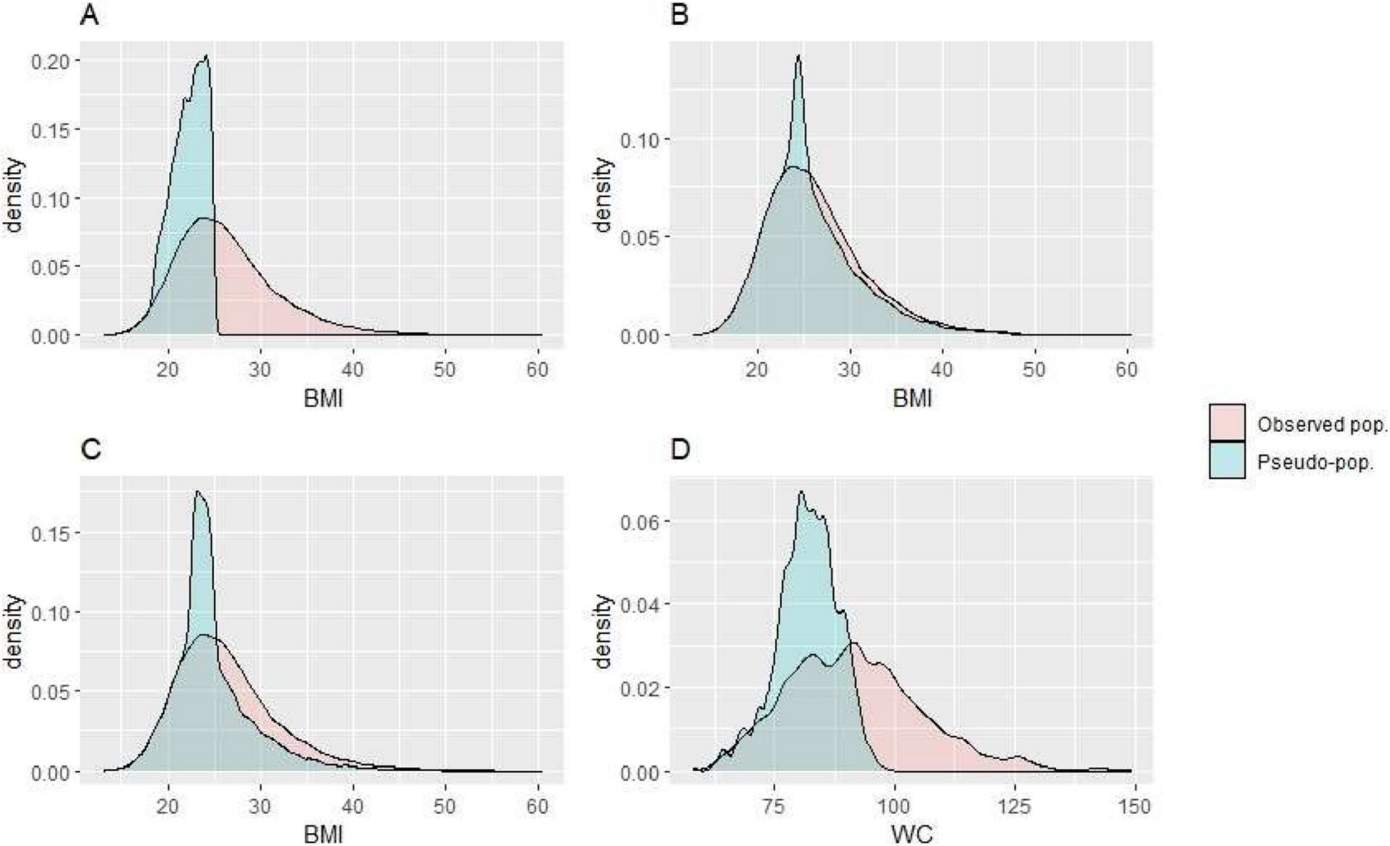
BMI and waist circumference distribution under the four weight-reduction scenarios

A total of 27,536 participants from the 2008, 2013, and 2018 Belgian Health Interview Surveys (BHIS), aged 18 years and above, were included in the analysis, with 1,184 of them participating in the 2018 Belgian Health Examination Survey (BELHES).The missing data pattern and summary statistics of all considered variables in the merged BHIS/BELHES dataset are displayed in Additional files 2–6. The impact of the four weight reduction scenarios on the BMI and WC distribution are visualized in Fig. 3.

### Association between excess weight and diabetes, hypertension, CVD, and MSK disease

Results of the multivariable logistic regression models showed a significant association between both BMI and WC and each of the four NCDs that were considered (Table 2). A stronger association was found for diabetes and hypertension compared to CVD and MSK disease. The four models for diabetes, hypertension, CVD, and MSK demonstrated a good predictive performance with AUC of 77%, 80%, 80%, and 72%, respectively. Forest plots of the logistic regression models for each NCD are displayed in Additional files 7, 8, 9, 10. The results of the sensitivity analysis without adjustment for physical activity showed similar estimates (additional file 11).

**Table 2:** Estimates of the logistic regression models (associations between waist circumference and BMI and diabetes, hypertension, cardiovascular disease, and musculoskeletal disease) and potential impact fractions for four scenarios; (1): distribution shift, (2): 1 point BMI reduction, (3): 10% reduction of weight, (4): waist circumference = height/2

		Diabetes		Hypertension		Cardiovascular disease		Musculoskeletal disease	
Baseline prevalence (%) [95% CI]		5.3 [4.7;6.0]		29.0 [27.5;30.5]		6.8 [6.4;7.2]		35.5 [34.8;36.2]	
OR (BMI) [95% CI] (for 1 IQR increase)		1.6 [1.4;1.8]		1.9 [1.6;2.1]		1.1 [1.0;1.2]		1.2 [1.1;1.3]	
OR (WC) [95% CI] (for 1 IQR increase)		1.9 [1.6;2.4]		2.0 [1.7;2.4]		1.2 [1.0;1.4]		1.2 [1.1;1.3]	
Target pop		BMI > 25	BMI > 30	BMI > 25	BMI > 30	BMI > 25	BMI > 30	BMI > 25	BMI > 30
(1)	RD (%) [95% CI]	-1.7 [-2.1;-1.3]	-1.2 [-1.5;-0.9]	-6.8 [-8.1;-5.6]	-4.4 [-5.2;-3.6]	-0.6 [-1.1;-0.2]	-0.4 [-0.7;-0.1]	-2.1 [-3.0;-1.3]	-1.3 [-1.9;-0.8]
	PIF (%) [95% CI]	32.3 [25.2;39.3]	22.9 [17.4;28.4]	23.3 [9;27.6]	15 [12.1;17.9]	9 [2.4;15.5]	5.9 [1.5;10.4]	6 [3.6;8.5]	3.8 [2.2;5.3]
(2)	RD (%) [95% CI]	-0.3 [-0.5;-0.1]	-0.1 [-0.2;-0.1]	-0.8 [-1.3;-0.4]	-0.4 [-0.4;-0.3]	-0.1 [-0.2;-0.0]	-0.04 [-0.1;0.0]	-0.3 [-0.4;-0.2]	-0.1 [-0.2;-0.1]
	PIF (%) [95% CI]	4.5 [3.4;5.7]	2.4 [1.7;3.1]	3.3 [2.2;4.3]	1.3 [0.8;1.8]	1.5 [0.9;3.8]	0.5 [0.1;0.9]	0.8 [0.5;1.1]	0.3 [0.2;0.4]
(3)	RD (%) [95% CI]	-0.7 [-0.9;-0.5]	-0.4 [-0.6;-0.3]	-2.7 [-3.2;-2.1]	-1.3 [-1.5;-1.0]	-0.2 [-0.4;-0.1]	-0.1 [-0.2;-0.0]	-0.8 [-1.2;-0.5]	-0.4 [-0.5;-0.2]
	PIF (%) [95% CI]	13.6 [10.3;16.9]	8.0 [5.8;10.3]	9.2 [7.3;11]	4.4 [3.5; 5.3]	3.5 [-0.9;6.1]	1.8 [0.4;3.1]	2.4 [1.4;3.3]	1.1 [0.6;1.6]
Target pop		WC:height ratio > 0.5							
(4)	RD (%) [95% CI]	-2.1 [-3.1;-1]		-7.2 [-8.6;-6]		-0.7 [-1.3;-0.2]		-2.5 [-3.3;-1.7]	
	PIF (%) [95% CI]	36.4 [27.5;47.3]		24.7 [20.6;28.9]		10.8 [3.3;18.3]		7.1 [4.7;9.4]	

*RD* Risk difference, *PIF* Potential impact fraction, *BMI* Body mass index, *CI* Confidence interval, *IQR* interquartile range

### Potential impact fractions of the four weight reduction scenarios

**Fig. 4:**
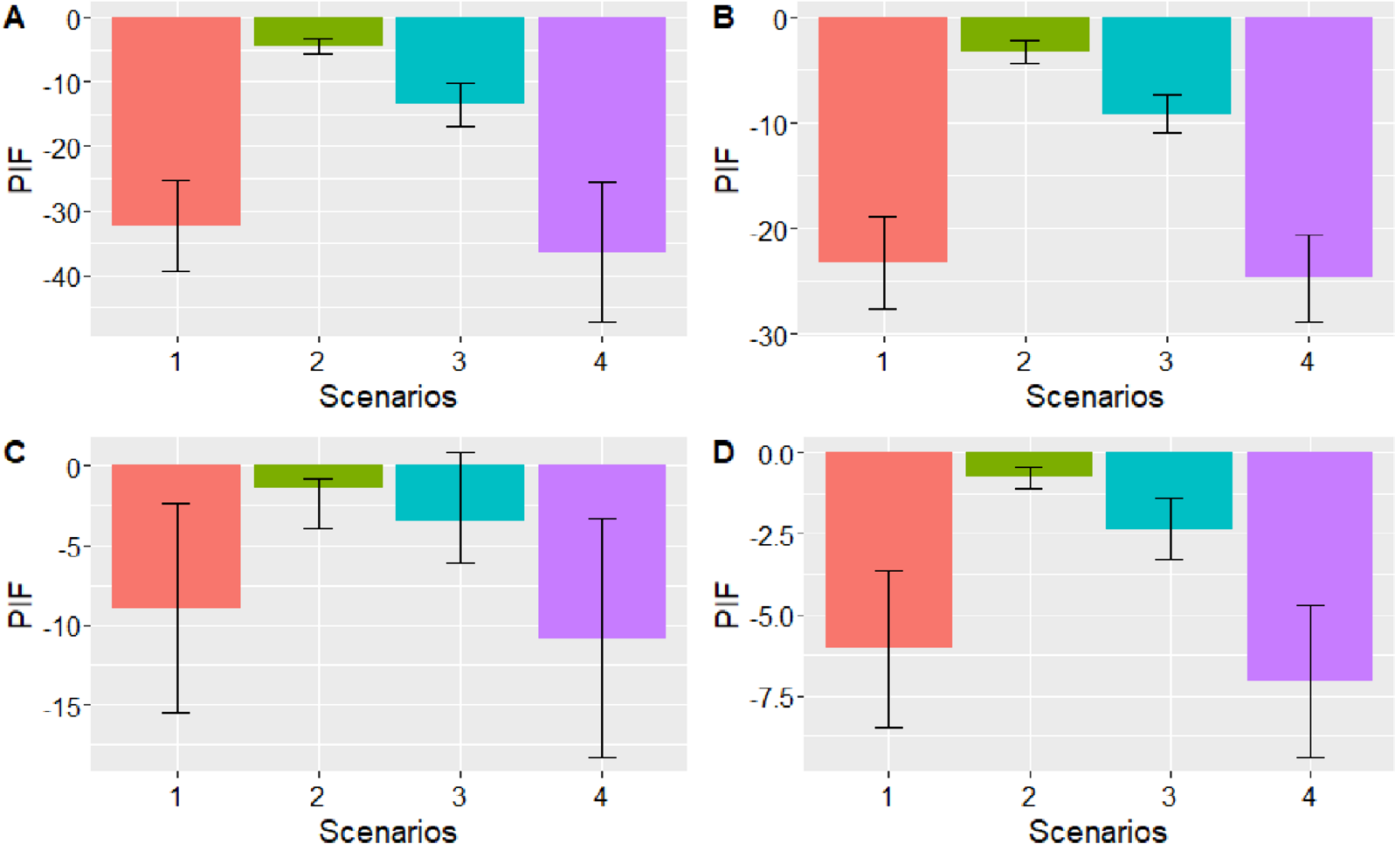
Bar plots illustrating the potential impact fraction (PIF) of the four weight reduction scenarios (1. distribution shift where, for each person with overweight, a counterfactual weight was drawn from the distribution of persons with a “normal” BMI, 2. One-unit reduction of the BMI of individuals with overweight, 3. modification of the BMI of individuals with overweight based on a weight loss of 10%, 4. reduction of the WC to the half of the height among all people with a WC/height ratio greater than 0.5) on **A**. diabetes, **B**. hypertension, **C**. cardiovascular disease, and **D**. musculoskeletal disease in Belgium. Error bars represent the 95% confidence intervals

The PIFs of the four weight reduction scenarios on diabetes, hypertension, CVD, and MSK disease in Belgium are visualized in Fig. 4. The average BMI reduction under the first three scenarios are respectively 4.2, 1 and 1.6 units and the average WC reduction under the last scenario is 9.9 cm. These amount to less than 1 SD (the conditional SD of BMI, given all the covariates equals 5.3 units, and of WC equals 13.2 cm).

The fourth scenario, where the waist circumference was reduced to half of the height had the highest impact on the four diseases considered, with nearly one third of the diabetes cases and one fourth of the hypertension cases that could have been avoided in the Belgian population. By contrast, the second scenario, where the BMI of people with excess weight was reduced by one unit had only a marginal impact on the four diseases considered. PIF were higher when the scenarios applied to people with overweight compared to people with obesity only (Table 2). The PIFs were all significantly different from 0, except for scenarios 3 related to CVD.

## Discussion

### Main findings

In this study, we presented a g-computation approach to evaluate the potential impact of hypothetical weight reduction scenarios on the burden of four NCDs in Belgium. We examined what would be the risk of suffering from diabetes, hypertension, CVD, and MSK disease if we could manipulate the BMI or the WC of Belgian adults and set them to values determined by hypothetical scenarios. The predicted risk was then compared to the risk under the “status quo” scenario, where no intervention would be implemented to the population. This is in contrast with the estimates we would have obtained using traditional regression models which produced stratum-specific odds ratios.

Our findings suggest that implementing weight reduction scenarios among individuals with excess weight could lead to a substantial and statistically significant decrease in the prevalence of diabetes, hypertension, CVD, and MSK diseases in Belgium. A major benefit was found for the fourth scenario, where the WC was lowered to half of the height for all Belgians with a ratio WC:height ratio above 0.5. Under this scenario, the prevalence of diabetes and hypertension would be drastically reduced, with respectively 36% and 25% of avoidable cases. The reduction was less pronounced for CVD and MSK diseases with a PIF of respectively 11% and 7%. A recent guideline report from the National Institute for Health and Care Excellence (NICE) mentioned that a waist measurement of more than half of a person’s height was a better indicator of increased fat in the abdomen compared to BMI and could better predict the risk of developing NCDs such as type 2 diabetes or CVD [53]. BMI remains however a useful practical measure to define overweight and obesity but should be interpreted with caution especially among older people and adults with high muscle mass, since it is less accurate to determine body fatness in these groups [58].

High PIFs were also observed under the first scenario, where the distribution of the BMI of all people with overweight would be shifted to the distribution of the BMI of people fallen in the “normal” BMI category. While this scenario may not be highly realistic, it is nonetheless valuable in defining the boundaries within which realist policy interventions could have an impact. This very theoretical scenario has the advantage to estimate the global burden of excess weight on NCDs and is closest to traditional PAF which estimate the risk of disease with a complete removal of the risk factor in the population. Under this first scenario, PIF for diabetes, hypertension, CVD, and MSK disease were 32%, 23%, 9%, and 6%, respectively. Those estimates were however lower in comparison to the PAF estimates obtained from the last Global Burden of Disease (GBD) study where the PAFs attributable to high BMI in Belgium were respectively of 50% for diabetes, 20% for ischemic heart diseases, 25% for stroke, 7% for back pain, and 13% for osteoarthritis [59].

It must be noted that those estimates cannot directly be compared to the estimates presented in this article. The g-computation approach is tailored to our data by estimating, for each individual, the conditional probability of developing a chronic disease given the variables included in the model, and subsequently averaging it at the population level.

In contrast, the GBD study's PAF estimates consider the overall contribution of high BMI to diseases across the entire population. They are not calculated directly from the specific population but often rely on relative risk estimates from external studies. These differences in data sources, methodologies, and the underlying framework for estimating population-level burden versus individual causal effects make direct comparisons between the two sets of estimates complex.

In addition, the variables for CVD and MSK used in this study were constructed based on a group of diseases (Table 1), which is difficult to compare with the GBD estimates, where PAFs are calculated for each disease separately.

The second and third scenario, where the BMI was respectively reduced by one unit and modified based on a ten percent reduction of the person’s weight, represent more realistic scenarios but had a smaller impact on the prevalence of the four diseases. A weight loss of 5–10% is considered by guidelines from the UK and the USA a the minimum weight loss to be achieved to have a clinical impact on health outcomes [58, 60]. To achieve this goal, evidence-based interventions include dietary modifications, physical activity, psychological interventions, pharmacotherapy, and bariatric surgery, for individuals with severe obesity [61, 62]. There is substantial evidence demonstrating that these interventions not only contribute to weight loss but also have a statistically significant impact on reducing the risk of obesity-related outcomes [63]. A one-unit reduction in BMI within the Belgian population would result in a reduction of 4.5% of the cases of diabetes, 3% of the cases of hypertension, 1.5% of the cases of CVD, and 1% of the cases of MSK disorders.

### Strengths and limitations

An important strength of this study lies in the didactic application of the g-computation approach and the description of the steps required to estimate the population effect of a potential intervention in cross-sectional data. The methodological tool used in this present study, based on a g-computation approach and a random-forest multiple imputation method, allows the assessment of the potential effects of any well-defined intervention and targeting of any subgroup of interest, while also addressing the bias related to self-reported data and the missing data issue in health interview surveys. This paper contributes to familiarizing a public health audience with the g-computation approach enabling them to estimate policy-relevant effects of hypothetical health interventions. Compared to standard analytic techniques, the g-computation approach has the advantage to provide flexibility in simulating real world interventions. It enables modeling the impact of dynamic interventions, where different subjects can receive varying levels of the exposure under study, as well as joint interventions, where the values of multiple exposures can be modified simultaneously. Another additional benefit of the g-computation approach, lies in its ability to handle time-varying confounders (i.e., confounders whose value changes over time), especially in situations where there's treatment confounder feedback (i.e., when the confounder is affected by the exposure) [64]. However, the cross-sectional nature of the data in this study did not allow us to take full advantage of this benefit.

This study also represents the first application of the random-forest multiple imputation method to address the bias related to self-reported health and anthropometric data in the BHIS. This method has been recently identified as a more adequate approach for valid measurement error correction in comparison to regression calibration [37]. Whenever feasible, self-reported information from health interview surveys should be combined with objective information from health examination surveys to address the bias related to self-reported anthropometric data and therefore provide more accurate PIF. A second important strength of the present study is the consideration of the potential confounding role of the environmental factors in the association between excess weight and chronic diseases. In particular, the linkage of the BHIS data with objective environmental factors at the residential address of the participants provides a significant improvement on the state of the art, as most studies do not consider environmental factors in the link between BMI and chronic diseases. Also, environmental factors are often assessed on a broad scale, using exposure e.g. in administrative units. Our study used the residential address, thus considerably refining the spatial scale. The limits of this approach are discussed further in the section *measurement error*.

Findings of this study must nevertheless be seen in the light of some limitations. If the g-computation approach allowed to evaluate the PIF of several weight reduction scenarios, the obtained estimates should however be treated with caution and several assumptions need to be met to interpret them causally. The first assumption is the “temporal ordering assumption” where we assume that the exposure precedes the outcome and the confounding factors precede the exposure. Unfortunately, this required assumption is not met by the cross-sectional structure of the data and is undoubtedly the most questionable assumption in this present study. While we can reasonably assume that fixed variables such as age, sex or education are causes rather than effects of the excess weight risk factor, it is not that obvious that the excess weight risk factor precedes chronic disease or that lifestyle factors precede the weight status. Making the distinction between unintentional weight loss, which may result from chronic disease, and intentional weight loss can be challenging [65]. People suffering from chronic disease could also be physically less active and therefore be at greater risk of gaining weight. For instance, individuals with CVD, MSK disorders or diabetes may exhibit weight gain due to factors like reduced mobility (leading to a decrease in calorie expenditure), medications, or fluctuations in blood sugar levels. Another challenge with cross-sectional data is the inability to differentiate whether covariates function as mediators or confounders. In this study, physical activity was considered as a confounding factor but it cannot be ruled out that excess weight may impact physical activity and indirectly the risk of chronic disease. One possible consequence could be underestimation of the true causal effect because the PAF would not incorporate all burden for the disease that is attributable to the excess weight risk factor. Physical activity could also function as a collider variable (a variable that is a common effect of both the exposure and the outcome) and adjusting for it may have introduced collider bias, potentially generating a spurious association between excess weight and chronic diseases.

The second assumption is the “exchangeability” assumption which assumes that there are no unmeasured confounding factors in the exposure-outcome association. Indeed, the exposure may only be considered as randomized within each stratum of the confounders if all confounders are considered in the model. This assumption is also very difficult to meet in the available cross-sectional study. Although we included in our analyses all the confounders identified in the literature that were available in our data, there remain several potential unmeasured confounding factors, such as genetic factors or nutritional habits which can both play an important role in the association between excess weight and chronic disease. Even though the variables related to nutritional habits were available in the BHIS, it was decided to not include them in the model because they were highly prone to a reverse causation effect.

The third assumption, known as the “no-interference assumption”, asserts that the outcome of each individual is not affected by the exposures and outcomes of the other individuals. We can reasonably expect that this assumption is fully met in our study for the reason that chronic diseases are not contagious. This, however, may vary depending on the intervention and study group. For instance, the implementation of a dietary intervention to reduce BMI of participants, such as changing the cooking style in the family, could potentially influence members of the same family similarly.

The fourth assumption, the “experimental treatment assignment” assumption, also called the positivity assumption [66], assumes that the exposure to the risk factor is possible for all individuals in each stratum of the covariates. In the context of this study, it means that the BMI values generated under the considered scenarios must be attainable for all individuals in which the scenario took place. This assumption is closely related the realism of the scenario and is therefore more likely violated for the first and fourth scenarios, which requires changes in the BMI or in the WC that are rarely observed in the population (e.g. a drop in the BMI from 35 to 25). In concrete terms, this means that each stratum of the covariates that contains overweight individuals should also contain individuals with a normal BMI. To evaluate the positivity assumption, we compared the probability of individuals being overweight among the two populations groups under study (individuals with overweight and individuals with a “normal” BMI). We built a model for BMI based on all confounders, and predicted, for each individual with overweight, the probability of being overweight. This process was repeated for individuals with a normal BMI. The observed overlap between the two probability distributions suggests that this assumption is plausible (Additional file 12).

The fifth assumption is the “consistency” assumption, which assumes that “an individual’s potential outcome under his observed exposure history is precisely his observed outcome” [19]. While consistency is plausible for medical treatments, because it is easy to manipulate hypothetically an individual's treatment status, consistency may however be problematic when the exposure is a biologic feature and the manipulation difficult to conceive [67]. Violations of consistency assumption often occur when there is ambiguity in the definition of interventions to change exposure. In the context of this study, BMI interventions remain vague because they specify attributes rather than specific behaviors. The main limitation of our approach lies in the highly theoretical nature of the hypothetical scenarios considered, which do not accurately mirror real-world interventions. Ambiguity arises from the fact that there are many competing approaches to decrease an individual’s BMI and each of these approaches may have a different causal effect on the outcome [68]. By presenting an estimate for the effect of a “BMI reduction”, we implicitly assume that all interventions on BMI have the same effect on the risk of suffering from a chronic disease, which is unlikely to hold. Another difficulty arising from ill-defined interventions is the challenge of selecting the confounding factors required to achieve conditional exchangeability. Firstly, the set of confounding factors to be considered may vary for different versions of the intervention. Secondly, because BMI is not an intervention in itself but rather a physiological risk factor, identifying all the confounders becomes a practically impossible task due to the necessity of also considering genetic factors. Even if we manage to account for all potential confounding factors including genetic factors, there is a high likelihood that the positivity assumption will be violated. Certain genetic traits could exert such a strong influence on body weight that all subjects possessing them automatically become obese [68]. Another issue with interventions on BMI is that the better we adjust for confounders that determine both excess weight and chronic diseases, the more we narrow our focus to the remaining factors that have a direct effect on BMI (such as genetic predispositions). Consequently we isolate a potential intervention that changes the remaining determinants of BMI. In this study, we compared the risk of suffering from a chronic disease of overweight vs non overweight individuals conditional on their physical activity level, smoking status, environmental and alcohol consumption. This means that our estimates correspond to the effect of other versions of the intervention “BMI reduction”, such as healthy diet or genes. However, other versions of the intervention may not be manipulable and not be of primary interest for policymakers. Successful interventions with evidence for effective weight reduction are multifactorial and it is unrealistic to assume that BMI in the population could be modified without considerable changes to all other aspects of lifestyle. Our findings may therefore be underestimated, since our analyses adjusted for possible confounding by physical activity or alcohol consumption and thereby do not entirely take into account the co-benefits of weight reduction intervention via changes in physical activity or alcohol consumption.

The sixth underlying assumption of g-computation approach is the “no model misspecification” assumption. A necessary condition (but not sufficient) for the absence of model misspecification is that the model should be able to accurately predict the outcome under no intervention. Variables from the model were selected based on their theoretical relevance and guided by a DAG that reflects the hypothesized causal structure. Non-linear relationships were assessed by testing the quadratic terms, while interactions were examined using the StepAIC algorithm (a variable selection method that iteratively adds or removes variables from a model based on their impact on the Akaike Information Criterion, aiming to find the most parsimonious model with a good fit). The AUC demonstrated a good predictive performance for the four NCDs models.

Lastly, like other studies based on observational data, the validity of our results relies on the key assumption of no measurement error. It can however be challenging to accurately assess the exposure to risk factors of NCDs through observational studies, such as abdominal obesity or environment. Although we applied a correction method to address the bias of self-reported anthropometric data and used both BMI and waist circumference separately to approximate abdominal obesity, another measure that could have been used is the Body Shape Index (ABSI), a comprehensive indicator of body shape integrating both waist circumference and BMI [69]. For the environment also, it is important to keep in mind that air pollution exposure is extrapolated from the mean annual concentration of a given area to individual exposure, and does not take into account the time spent in this area. Personal mobility could be integrated in dynamic exposure assessments, but determining individual buffer values to delimit a person’s neighborhood is still an active field of research. Other methods to determine environmental exposure are human biomonitoring or deploying wearable sensors, but this is unfortunately impossible to apply for large samples, over long time periods or for past studies. There was also a time lag between health data collection and environmental data. However, as environmental change is slow, we do not expect a strong impact on our results. A certain degree of measurement error also applies for the diseases. While the bias related to self-reported diabetes and hypertension could be addressed based on clinical information from the BELHES, the same correction could not be applied for self-reported CVD and MSK diseases, as the relevant clinical information was not available in the BELHES.

Furthermore, our estimates apply to the Belgian population and may not be generalizable to other populations characterized by different NCDs risk factor distributions. For example, we estimated the risk of diabetes in the Belgian population for a distribution shift of the BMI of individuals with overweight to the distribution of individuals with a normal BMI, but the BMI distribution may be very different in other populations. Our PIF estimates may also vary a lot for different diseases within the same CVD or MSK group, limiting the possibility of comparing our results with the GBD estimates.

A final limitation of our study lies the lack of detailed analysis regarding the differential effects of BMI on different types of diabetes. While our findings demonstrate a significant association between BMI and diabetes, it must be recognized that the impact of BMI may vary between type 1 and type 2 diabetes. While the link between obesity and type 2 diabetes is well-established, emerging evidence suggests a link between obesity and type 1 diabetes as well [70]. Future research could explore this aspect further to elucidate whether BMI affects both types of diabetes similarly.

Whilst obesity is widely considered as a major modifiable risk factor for many chronic diseases, nevertheless, a rigorous examination of the mentioned assumptions underscores the challenge in determining its causes and consequences. Addressing this is however important, as the prevention of any disease requires that interventions focus on causal risk factors. Although all the required assumptions of the g-computation approach may not be fully met, based on the literature knowledge regarding the relationship between excess weight and NCDs, the evidence from literature supports the direction of causality investigated in this study.

## Conclusions

This study gives a demonstration of the use of a g-computation approach to assess the benefits of hypothetical weight reduction scenarios on NCDs in Belgium in a multi-exposure context. Results suggest that implementing weight reduction scenarios among individuals with excess weight could lead to a substantial and statistically significant decrease in the prevalence of diabetes, hypertension, cardiovascular disease (CVD), and musculoskeletal (MSK) diseases in Belgium. The g-computation based approach to assess PIF of interventions represents a straightforward approach in epidemiology for making causal inference from observational data while providing also useful information for policy makers. Future epidemiological and health impact assessment studies should be conducted in ways that are more informative for policymakers and should consider all the underlying assumptions explicitly in order to better evaluate the possibility of a causal effect. In particular, we acknowledge the importance of the consistency assumption in ensuring the validity of the study’s findings, especially within the field of obesity epidemiology. Ideally, longitudinal studies including time-varying data should be used in the future to address the “temporal ordering assumption” in the association between excess weight and chronic diseases.

### Supplementary Information

**Supplementary Material 1.**

**Supplementary Material 2.**

**Supplementary Material 3.**

**Supplementary Material 4.**

**Supplementary Material 5.**

**Supplementary Material 6.**

**Supplementary Material 7.**

**Supplementary Material 8.**

**Supplementary Material 9.**

**Supplementary Material 10.**

**Supplementary Material 11.**

**Supplementary Materail 12.**

## Data Availability

The data that support the findings of this study are not publicly available. Data are however available from the authors upon reasonable request and with specific permission (https://www.sciensano.be/en/node/55737/health-interview-survey-microdata-request-procedure). Legal restrictions make that BHIS and BELHES data can only be communicated to other parties if an authorization is obtained from the sectoral committee social security and health of the Belgian data protection authority.
