# Supplementary material for: The potential impact fraction of population weight reduction scenarios on non-communicable diseases in Belgium: application of the g-computation approach
Source: BMC Med Res Methodol. 2024 Apr 14;24:87. doi: 10.1186/s12874-024-02212-7 (PMC11016220; doi:10.1186/s12874-024-02212-7)
Supplement: Supplementary file 1 — Supplementary Material 1. [file 12874_2024_2212_MOESM1_ESM.pdf]

Additional file 1: missing data pattern for socio-economic and lifestyle variables of the merged dataset BHIS/BHES

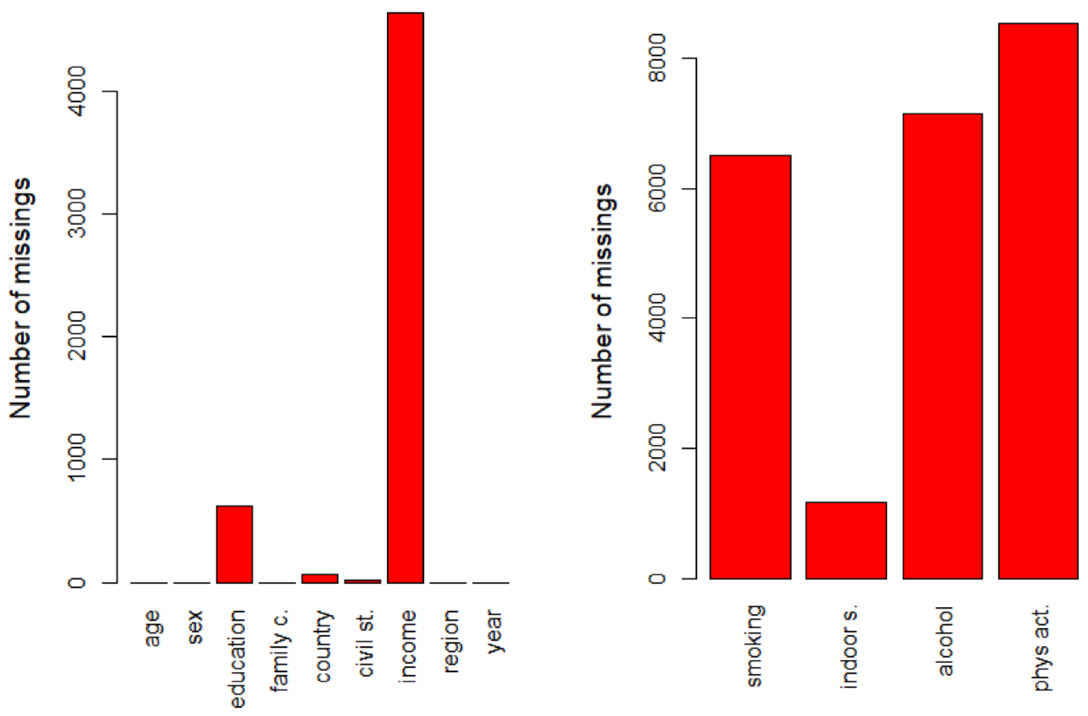

SR: self-reported, HT: hypertension, M: measured, WC: waist circumference, Myocardial: myocardial infarction, anginapect: angina pectoris, peri.vasc.dis.: peripheral vascular diseases, other_heart: other serious heart diseases, back: low back pain, neck: neck pain, osteoarth: osteoarthritis, rharthrosis: rheumatoid arthritis
