# Supplementary material for: The potential impact fraction of population weight reduction scenarios on non-communicable diseases in Belgium: application of the g-computation approach
Source: BMC Med Res Methodol. 2024 Apr 14;24:87. doi: 10.1186/s12874-024-02212-7 (PMC11016220; doi:10.1186/s12874-024-02212-7)
Supplement: Supplementary file 3 — Supplementary Material 3. [file 12874_2024_2212_MOESM3_ESM.pdf]

Additional file 3: missing data pattern for environmental variables of the merged dataset BHIS/BHES

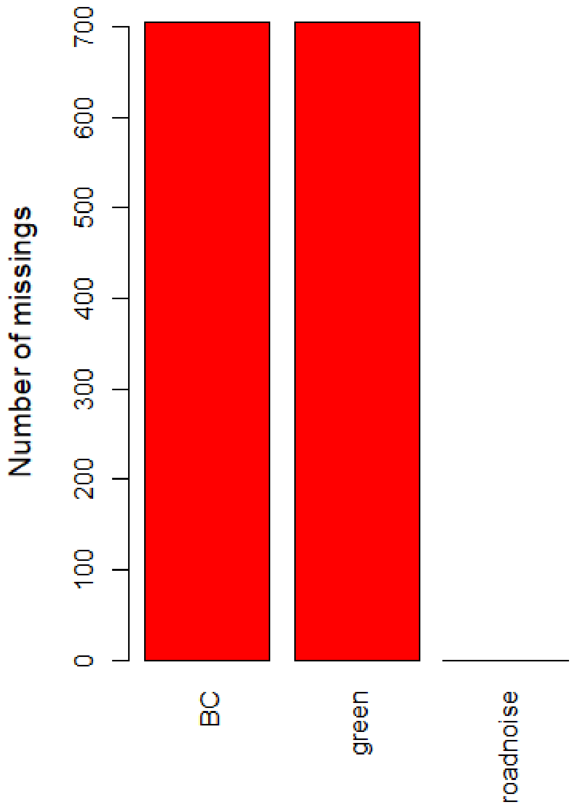

BC: Black carbon, Green: green coverage in 1 km buffer, Roadnoise: noise from the road traffic
