# Supplementary material for: The potential impact fraction of population weight reduction scenarios on non-communicable diseases in Belgium: application of the g-computation approach
Source: BMC Med Res Methodol. 2024 Apr 14;24:87. doi: 10.1186/s12874-024-02212-7 (PMC11016220; doi:10.1186/s12874-024-02212-7)
Supplement: Supplementary file 4 — Supplementary Material 4. [file 12874_2024_2212_MOESM4_ESM.pdf]

Additional file 4: Description of the merged BHIS/BELHES dataset

	Proportion (%) [95% CI]	Mean [SE]	Median [IQR]	n
**Anthropometric measures**				
SR. height (cm)		170.35 [0.08]	170 [13]	27150
M. height (cm)		169.76 [0.38]	169 [14]	1181
SR. weight (kg)		71.46 [0.11]	70 [18.5]	27259
M. weight (kg)		76.76 [0.57]	74.7 [21.7]	1179
SR. BMI (kg/m^2)		24.77 [0.04]	24.4 [5.21]	25061
M. BMI (kg/m^2)		26.61 [0.19]	25.75 [6.34]	1179
M. waist circumference		92.31 [0.54]	91.5 [19]	1172
**Non-communicable diseases**				
SR. diabetes				27428
Yes	5.38 [5.04;6]			1711
No	94.6 [94.2;95]			25717
M. diabetes				1062
Yes	10.14 [8.08;13]			105
No	89.9 [87.3;92]			957
SR. hypertension				27403
Yes	17.4 [16.7;18]			5049
No	82.6 [82;83]			22354
M. hypertension				1179
Yes	32.8 [29.4;36]			382
No	67.2 [63.5;71]			792
SR. cardiovascular diseases				18652
yes	8.13 [7.59;9]			1700
No	91.9 [91.3;92]			16952
SR. musculoskeletal disorders				27178
Yes	35.2 [34.4;36]			9589
No	64.8 [63.9;66]			17589
**Socio-economic status**				
Age (year)		49.08 [0.18]		27536
Sex				27536
Man	48.3 [47.7;49]			12930
Women	51.7 [51.2;52]			14606
Education level				26922
No diploma/Prim	9.71 [9.13;10]			3154
Low secondary	14 [13.3;15]			3979
High secondary	33.2 [32.2;34]			8348
Higher	43.1 [41.9;44]			11441
Country of birth				27509
Belgian	84.6 [84;85]			21415
Non belgian EU	6.74 [6.32;7]			2824
Non belgian non EU	8.64 [8.12;9]			3232
Family composition				27536
Single	19.2 [18.5;2]			6504
One parent with child(ren)	8.09 [7.52;9]			2294
Couple without child(ren)	27.6 [26.6 ;29]			7214
Couple with child(ren)	37.1 [36.0;38]			9233
Other or unknown	7.98 [7.33;9]			2291
Civil status				27515
Single	27.1 [26.3;28]			7256
Maried	55.7 [54.8;57]			14691
Widow	7.79 [7.39;8]			2873

Divorced	9.4 [8.91;10]			2695
**Lifestyle**				
Physical activity				19000
Sport > 4 houres/week	16.7 [16;0.18]			2897
Sport < 4 houres/week	54.4 [53.4;55]			10102
Sedentary	28.9 [28;3]			6001
Smoking status				21024
Daily smokers	18.6 [17.8;19]			3866
Occasional smokers	4.02 [3.67;4]			889
Former smokers	22.9 [22.1;24]			4735
Never smoked	54.5 [53.5;55]			11534
Indoor smoking				26372
Yes	21.1 [20.2;22]			5853
No	78.9 [78;8]			20519
Alcohol consumption		1.95 [0.01]	2 [1]	20380
**Environment**				
Black carbon exposure (µg/m^3)		1.16 [0.05]	1.10 [0.57]	26831
Vegetation coverage (1km buffer)		40.34 [0.3]	30.8 [55.1]	26831
Road noise (Lden)				27536
>55dB	11.6 [10.8;12.1]			2634
<55dB	88.4 [87.6;89]			24902
**Region**				
Brussel's region	10.6 [10.4;0.11]			7631
Walloon's region	32.2 [31.6;33]			10187
Flemish region	57.1 [56.4;58]			9718
**Year**				
2008	31.3 [30.3;32]			
2013	32.8 [31.7;34]			
2018	35.9 [34.8;37]			

M: Measured, SR: self-reported
