# Supplementary material for: The potential impact fraction of population weight reduction scenarios on non-communicable diseases in Belgium: application of the g-computation approach
Source: BMC Med Res Methodol. 2024 Apr 14;24:87. doi: 10.1186/s12874-024-02212-7 (PMC11016220; doi:10.1186/s12874-024-02212-7)
Supplement: Supplementary file 5 — Supplementary Material 5. [file 12874_2024_2212_MOESM5_ESM.pdf]

Additional file 5. Description of the study population after random-forest multiple imputation (estimates obtained using Rubin's Rules)

	Proportion (%) [95% CI]	Mean [SE]	Median [IQR]	N=27536
**Anthropometric measures**				
height (cm)		169.23 [0.07]		
weight (kg)		75.02 [0.13]		
BMI (kg/m^2)		26.23 [0.04]	25.51 [6.62]	
waist circumference		91.21 [0.12]	91 [18.8]	
**Non-communicable diseases**				
diabetes				
Yes	4.56 [4.01;5.11]			
hypertension				
Yes	26.8[25.25;28.34]			
cardiovascular diseases				
yes	6.13 [5.71;6.54]			
musculoskeletal disorders				
Yes	35 [34.1;36]			
**Socio-economic status**				
Age (year)		49.08 [0.18]		
Sex				
Man	51.7 [51.2;52]			
Women	48.3 [47.7;49]			
Education level				
No diploma/Prim	9.77 [9.13;10]			
Low secondary	14 [13.54;14.45]			
High secondary	33.2 [29.8;36.8]			
Higher	43 [41.8;44.1]			
Country of birth				
Belgian	84.6 [83.93;85.2]			
Non belgian EU	6.73 [6.31;7.14]			
Non belgian non EU	8.63 [8.12;9.13]			
Family composition				
Single	19.2 [18.61;19.78]			
One parent with child(ren)	8.09 [7.54;8.63]			
Couple without child(ren)	27.6 [26.62;28.58]			
Couple with child(ren)	37.1 [35.92;38.27]			
Other or unknown	7.98 [7.39;8.57]			
Civil status				
Single	27.1[26.31;27.88]			
Maried	55.7 [54.9;56.48]			
Widow	7.79 [7.39;8.18]			
Divorced	9.41 [9.01;9.80]			
**Lifestyle**				
Physical activity				
Sport > 4 houres/week	15.6 [15.01;16.18]			
Sport < 4 houres/week	54.8 [53.85;55.7]			
Sedentary	29.6 [28.61;30.18]			

Smoking status				
Daily smokers	18.5 [17.8;19.2]			
Occasional smokers	3.76 [3.44;4.07]			
Former smokers	21.2 [20.49;21.90]			
Never smoked	56.51 [55.9;57.4]			
Indoor smoking				
Yes	21.1 [19.92;21.6]			
No	79.2 [78.29;80.1]			
Alcohol consumption		1.89 [0.01]		
**Environment**				
Black carbon exposure (µg/m^3)		1.17 [0.05]		
Vegetation coverage (1km buffer)		40.17 [0.03]		
Road noise (Lden)				
>55dB	11.6 [10.89;12.3]			
<55dB	88.4 [87.64;89.1]			
**Region**				
Brussel's region	10.6 [10.36;10.84]			
Walloon's region	32.2 [31.6;32.9]			
Flemish region	57.1 [56.4;57.8]			
**Year**				
2008	31.3 [30.27;32.32]			
2013	32.8 [31.74;33.8]			
2018	35.9 [34.82;36.97]			
