# Supplementary material for: The potential impact fraction of population weight reduction scenarios on non-communicable diseases in Belgium: application of the g-computation approach
Source: BMC Med Res Methodol. 2024 Apr 14;24:87. doi: 10.1186/s12874-024-02212-7 (PMC11016220; doi:10.1186/s12874-024-02212-7)
Supplement: Supplementary file 6 — Supplementary Material 6. [file 12874_2024_2212_MOESM6_ESM.pdf]

Additional file 6. R code implementation of the g-computation approach to assess the Potential Impact Fraction (PIF) for the first scenario (BMI distribution shift) on the risk of diabetes.

```
library(boot)
library(survey)
library(dplyr)

#FIRST DATASET- SCENARIO 1
# Define function for RD1_SCENARIO1

normalbmi <-  filter(RFDS1,BMI_M <25 & BMI_M >=18.5)

RD1_SCENARIO1 <-function (formula, data, indices) {
  d<-RFDS1 [indices,]
  RFDS1_SCENARIO<-RFDS1<-d
  RFDS1_SCENARIO$BMI_M[RFDS1_SCENARIO$BMI_M>=25] <- sample (normalbmi$BMI_M,size=
sum(RFDS1$BMI_M>=25), replace = T)
  sample_design <- svydesign(id = ~hh_cluster, strata = ~provw , data = d ,weights = ~wfin)
  mod1=svyglm (formula =M_diabetes~ age + sex + education + comp+ civstat + country  +income + smoking +
indoorsmoking + alcohol + physact + BC  + green_1000+road_noise2+ year + region +
BMI_M,family=quasibinomial(link=logit),design= sample_design, data=d)
  p_intervention <- predict(mod1, newdata= RFDS1_SCENARIO, type="response")
  p_population <- predict(mod1, newdata= RFDS1, type="response")
  meanY1 <-mean (p_intervention)
  meanY2 <-mean (p_population)
  AY1 <-mean (p_population) -mean (p_intervention)
  PIF<-AY1/meanY2
  return (list=c(AY1,meanY1,meanY2,PIF))
}

#Bootstrap for risk difference (RD) and PIF calculations

result1_1 <- boot(formula=mod1, data=RFDS1, statistic= RD1_SCENARIO1, R=1000)

# Extract and store relevant statistics (Risk difference & PIF)

RD_DATA1_SCENARIO1<- result1_1$t0[1]
SDRD_DATA1_SCENARIO1<- summary(result1_1)$bootSE[1]
VARRD_DATA1_SCENARIO1 <- (SDRD_DATA1_SCENARIO1)^2

PIFscenario_DATA1_SCENARIO1 <-result1_1$t0[4]
SDPIFscenario_DATA1_SCENARIO1 <-summary(result1_1)$bootSE[4]
VARPIFscenario_DATA1_SCENARIO1 <- (SDPIFscenario_DATA1_SCENARIO1)^2


#This analysis is then repeated in each of the ten completed datasets (obtained with multiple imputation). Code
provided for first dataset analysis; Repetition in remaining nine datasets omitted for brevity.

#RISK DIFFERENCE OF DIABETES IN SCENARIO 1 # Pooled estimate

#RD SCENARIO 1
RD_SCENARIO1_diabetes_pooled <-mean (c(RD_DATA1_SCENARIO1, RD_DATA2_SCENARIO1,
RD_DATA3_SCENARIO1, RD_DATA4_SCENARIO1, RD_DATA5_SCENARIO1, RD_DATA6_SCENARIO1,
RD_DATA7_SCENARIO1, RD_DATA8_SCENARIO1, RD_DATA9_SCENARIO1, RD_DATA10_SCENARIO1))

RD_SCENARIO1_diabetes_pooled
```

```
#Rubin’s rule SE

#WITHIN VARIANCE
withinVAR_RD_SCENARIO1_diabetes_pooled <-mean (c(VARRD_DATA1_SCENARIO1,
VARRD_DATA2_SCENARIO1, VARRD_DATA3_SCENARIO1,VARRD_DATA4_SCENARIO1,
VARRD_DATA5_SCENARIO1, VARRD_DATA6_SCENARIO1,VARRD_DATA7_SCENARIO1,
VARRD_DATA8_SCENARIO1, VARRD_DATA9_SCENARIO1,VARRD_DATA10_SCENARIO1))

withinVAR_RD_SCENARIO1_diabetes_pooled

#BETWEEN VARIANCE
betweenVAR_RD_SCENARIO1_diabetes_pooled<- var(c(RD_DATA1_SCENARIO1, RD_DATA2_SCENARIO1,
RD_DATA3_SCENARIO1, RD_DATA4_SCENARIO1,RD_DATA5_SCENARIO1, RD_DATA6_SCENARIO1,
RD_DATA7_SCENARIO1, RD_DATA8_SCENARIO1, RD_DATA9_SCENARIO1, RD_DATA10_SCENARIO1))

betweenVAR_RD_SCENARIO1_diabetes_pooled

#TOTAL VARIANCE
TOTVAR_RD_SCENARIO1_diabetes_pooled <-(withinVAR_RD_SCENARIO1_diabetes_pooled)+ ((1 + 1/10)*
betweenVAR_RD_SCENARIO1_diabetes_pooled)

TOTVAR_RD_SCENARIO1_diabetes_pooled

##FINAL SD
SDRD_SCENARIO1_diabetes_pooled <-sqrt (TOTVAR_RD_SCENARIO1_diabetes_pooled )

SDRD_SCENARIO1_diabetes_pooled

RD_SCENARIO1_diabetes_pooled+(1.96*SDRD_SCENARIO1_diabetes_pooled)
RD_SCENARIO1_diabetes_pooled-(1.96*SDRD_SCENARIO1_diabetes_pooled)

#PIF SCENARIO 1# # Pooled estimate

PIF_SCENARIO1_diabetes_pooled<-mean (c(PIFscenario_DATA1_SCENARIO1,
PIFscenario_DATA2_SCENARIO1, PIFscenario_DATA3_SCENARIO1, PIFscenario_DATA4_SCENARIO1,
PIFscenario_DATA5_SCENARIO1, PIFscenario_DATA6_SCENARIO1,PIFscenario_DATA7_SCENARIO1,
PIFscenario_DATA8_SCENARIO1, PIFscenario_DATA9_SCENARIO1, PIFscenario_DATA10_SCENARIO1))

PIF_SCENARIO1_diabetes_pooled

##Rubin’s rule SE

#WITHIN VARIANCE
withinVAR_PIF_SCENARIO1_diabetes_pooled <-mean (c(VARPIFscenario_DATA1_SCENARIO1,
VARPIFscenario_DATA2_SCENARIO1, VARPIFscenario_DATA3_SCENARIO1,
VARPIFscenario_DATA4_SCENARIO1, VARPIFscenario_DATA5_SCENARIO1,
VARPIFscenario_DATA6_SCENARIO1,VARPIFscenario_DATA7_SCENARIO1,
VARPIFscenario_DATA8_SCENARIO1, VARPIFscenario_DATA9_SCENARIO1,
VARPIFscenario_DATA10_SCENARIO1))

withinVAR_PIF_SCENARIO1_diabetes_pooled

#BETWEEN VARIANCE
betweenVAR_PIF_SCENARIO1_diabetes_pooled<-var(c(PIFscenario_DATA1_SCENARIO1,
PIFscenario_DATA2_SCENARIO1, PIFscenario_DATA3_SCENARIO1, PIFscenario_DATA4_SCENARIO1,
PIFscenario_DATA5_SCENARIO1, PIFscenario_DATA6_SCENARIO1, PIFscenario_DATA7_SCENARIO1,
PIFscenario_DATA8_SCENARIO1, PIFscenario_DATA9_SCENARIO1, PIFscenario_DATA10_SCENARIO1))
```

```
betweenVAR_PIF_SCENARIO1_diabetes_pooled

#TOTAL VARIANCE
TOTVAR_PIF_SCENARIO1_diabetes_pooled <-(withinVAR_PIF_SCENARIO1_diabetes_pooled)+ ((1 + 1/10)*
betweenVAR_PIF_SCENARIO1_diabetes_pooled)

TOTVAR_PIF_SCENARIO1_diabetes_pooled

#FINAL SE
SDPIF_SCENARIO1_diabetes_pooled <-sqrt (TOTVAR_PIF_SCENARIO1_diabetes_pooled)
SDPIF_SCENARIO1_diabetes_pooled

#CI
PIF_SCENARIO1_diabetes_pooled - (SDPIF_SCENARIO1_diabetes_pooled *1.96)
PIF_SCENARIO1_diabetes_pooled + (SDPIF_SCENARIO1_diabetes_pooled *1.96)
```
