# Supplementary figures and images for: The potential impact fraction of population weight reduction scenarios on non-communicable diseases in Belgium: application of the g-computation approach
Source: BMC Med Res Methodol. 2024 Apr 14;24:87. doi: 10.1186/s12874-024-02212-7 (PMC11016220; doi:10.1186/s12874-024-02212-7)

# Additional file 7 . Forest plot of the logistic regression model for diabetes

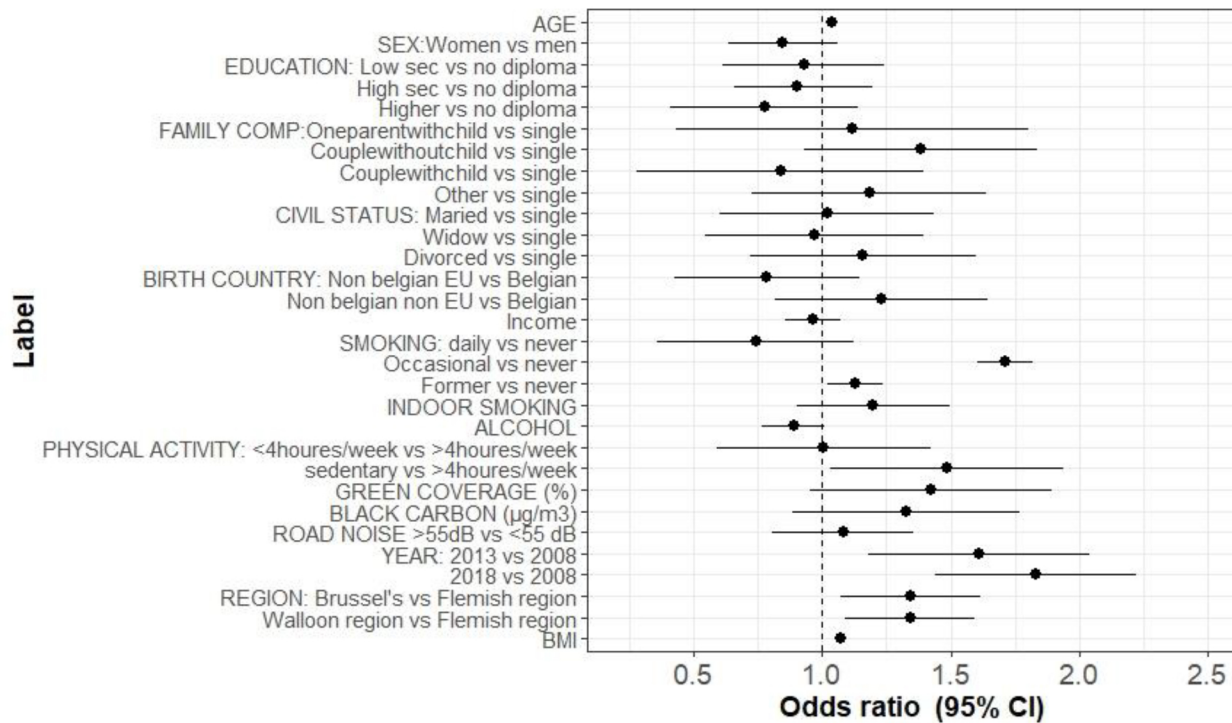

Supplement: Supplementary file 7 — Supplementary Material 7. [file 12874_2024_2212_MOESM7_ESM.pdf]

Additional file 8. Forest plot of the logistic regression model for hypertension

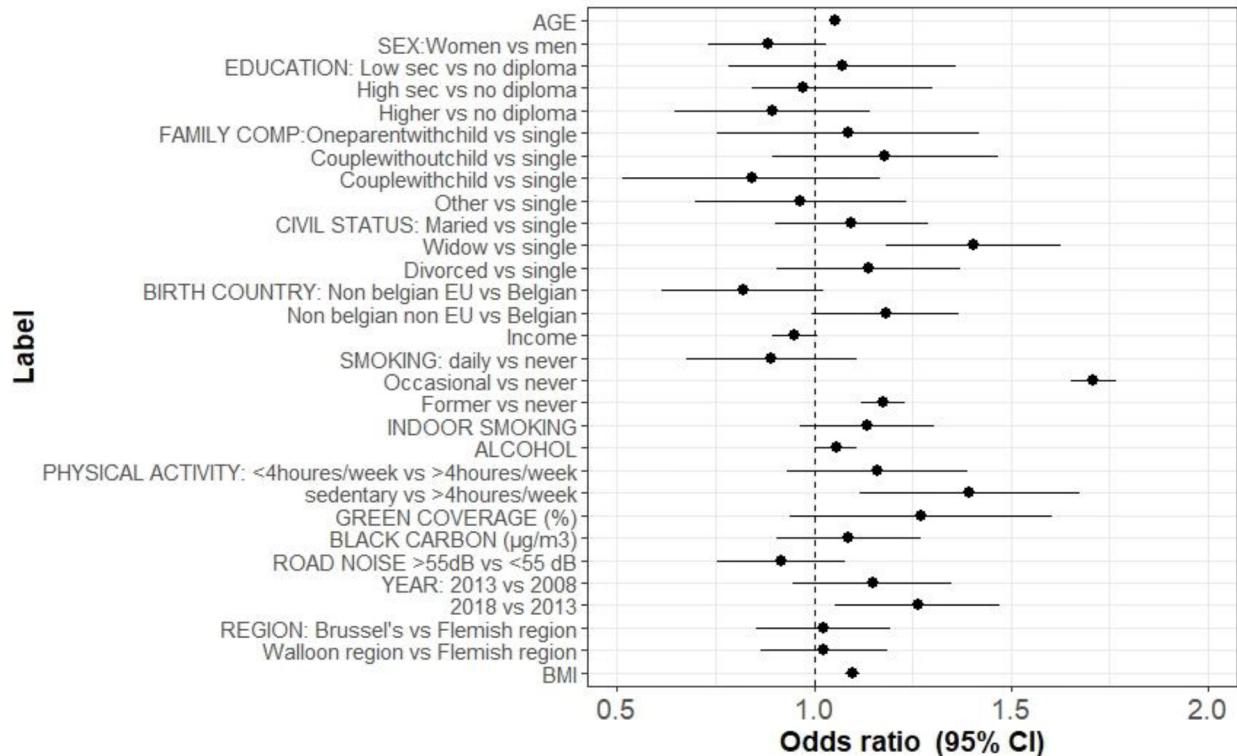

Supplement: Supplementary file 8 — Supplementary Material 8. [file 12874_2024_2212_MOESM8_ESM.pdf]

Additional file 9. Forest plot of the logistic regression model for cardiovascular diseases

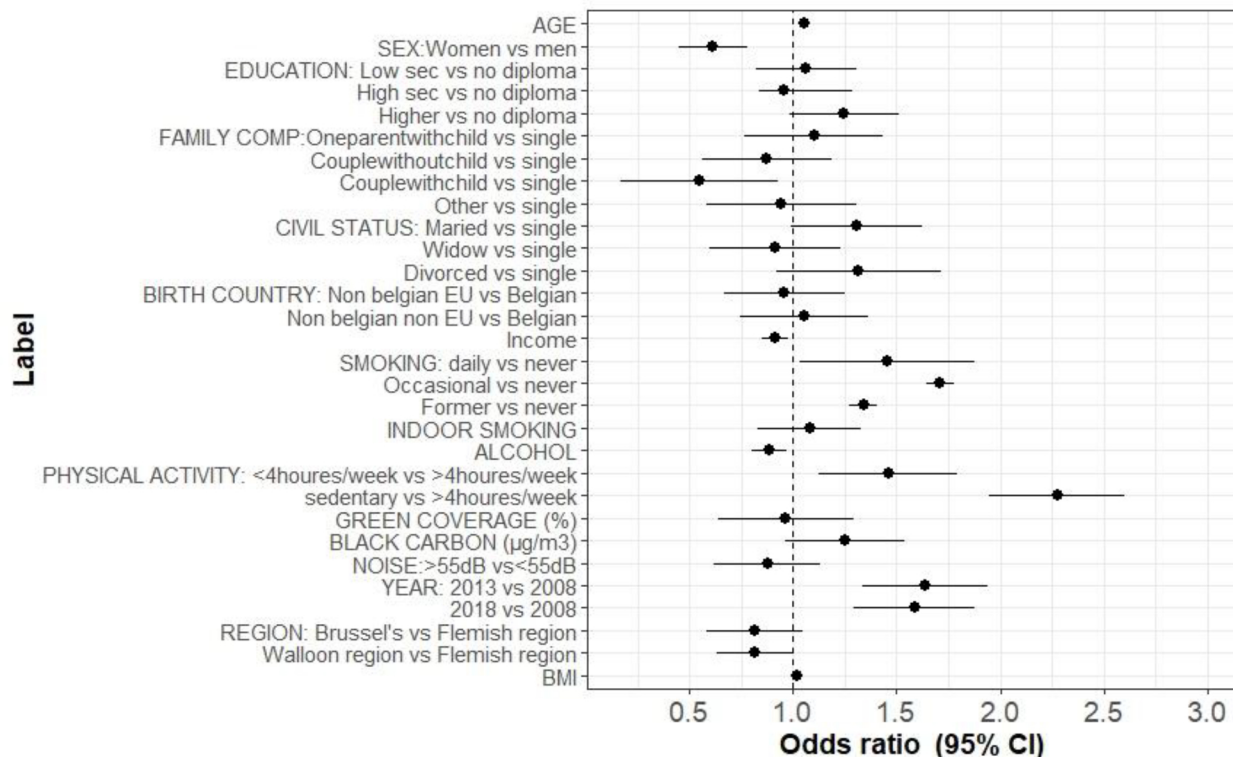

Supplement: Supplementary file 9 — Supplementary Material 9. [file 12874_2024_2212_MOESM9_ESM.pdf]

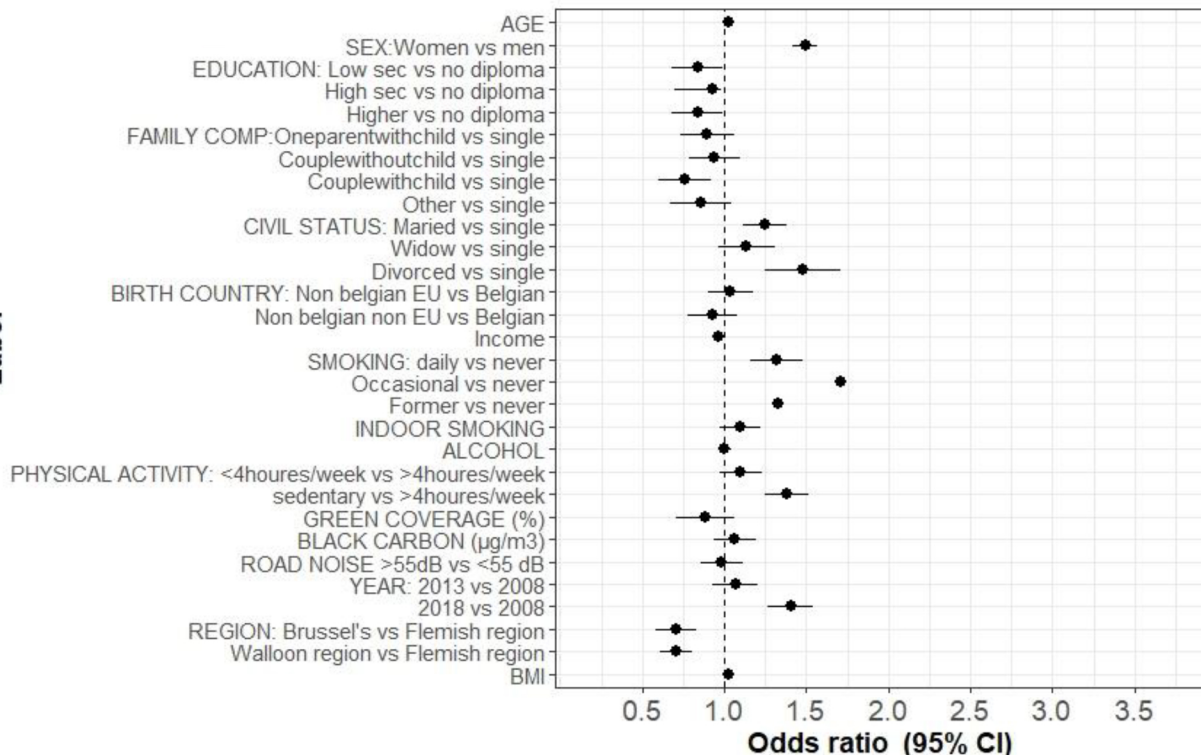

Supplement: Supplementary file 10 — Supplementary Material 10. [file 12874_2024_2212_MOESM10_ESM.pdf]
