# Supplementary material for: The potential impact fraction of population weight reduction scenarios on non-communicable diseases in Belgium: application of the g-computation approach
Source: BMC Med Res Methodol. 2024 Apr 14;24:87. doi: 10.1186/s12874-024-02212-7 (PMC11016220; doi:10.1186/s12874-024-02212-7)
Supplement: Supplementary file 11 — Supplementary Material 11. [file 12874_2024_2212_MOESM11_ESM.pdf]

Additional file 11. Estimates of the logistic regression models (association between waist circumference and BMI and diabetes, hypertension, cardiovascular diseases and musculoskeletal diseases) without adjustment for physical activity

	Diabetes	Hypertension	Cardiovascular disease	Musculoskeletal disease
OR (BMI)<br><br>[95% CI]<br>(1 IQR increase)	1.6<br><br>[1.4;1.8]	1.9<br><br>[1.7;2.1]	1.1<br><br>[1.0;1.2]	1.2<br><br>[1.1;1.3]
OR (WC)<br><br>[95% CI]<br>(1 IQR increase)	2<br><br>[1.6;2.4]	2.0<br><br>[1.7 ;2.4]	1.2<br><br>[1.0 ;1.4]	1.2<br><br>[1.1 ;1.3]

WC: Waist circumference, OR: odd ratio, IQR: interquartile range, CI: confidence interval, BMI: Body Mass Index
