# Supplementary material for: The potential impact fraction of population weight reduction scenarios on non-communicable diseases in Belgium: application of the g-computation approach
Source: BMC Med Res Methodol. 2024 Apr 14;24:87. doi: 10.1186/s12874-024-02212-7 (PMC11016220; doi:10.1186/s12874-024-02212-7)
Supplement: Supplementary file 12 — Supplementary Materail 12. [file 12874_2024_2212_MOESM12_ESM.pdf]

Additional file 12 : Distribution of the probability of being overweight among the population with a “normal” BMI (<25) and among the population with overweight (BMI >= 25).

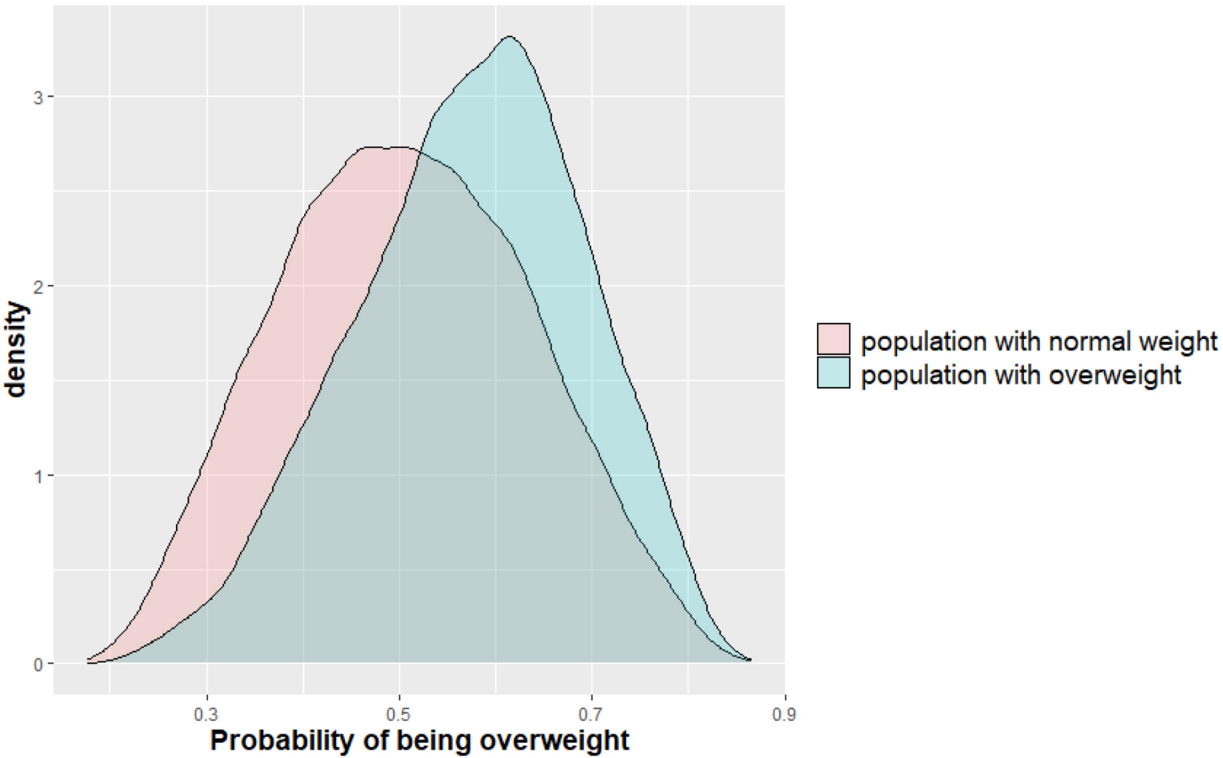

Predictions based on the logistic regression model Overweight~ age + sex + education + family composition + civil status + income + birth country + smoking + indoor smoking + alcohol + physical activity + green coverage + noise + year + region.
